# Transgenic insertion of the cyanobacterial membrane protein *ictB* increases grain yield in *Zea mays* through increased photosynthesis and carbohydrate production

**DOI:** 10.1371/journal.pone.0246359

**Published:** 2021-02-04

**Authors:** Robert P. Koester, Charles P. Pignon, Dylan C. Kesler, Rebecca S. Willison, Miyoung Kang, Yu Shen, Henry D. Priest, Matthew B. Begemann, Kevin A. Cook, Gary A. Bannon, Mohammed Oufattole

**Affiliations:** Benson Hill, St. Louis, Missouri, United States of America; Wageningen University, NETHERLANDS

## Abstract

The C_4_ crop maize (*Zea mays*) is the most widely grown cereal crop worldwide and is an essential feedstock for food and bioenergy. Improving maize yield is important to achieve food security and agricultural sustainability in the 21^st^ century. One potential means to improve crop productivity is to enhance photosynthesis. *ictB*, a membrane protein that is highly conserved across cyanobacteria, has been shown to improve photosynthesis, and often biomass, when introduced into diverse C_3_ plant species. Here, *ictB* from *Synechococcus* sp. strain PCC 7942 was inserted into maize using *Agrobacterium*-mediated transformation. In three controlled-environment experiments, *ictB* insertion increased leaf starch and sucrose content by up to 25% relative to controls. Experimental field trials in four growing seasons, spanning the Midwestern United States (Summers 2018 & 2019) and Argentina (Winter 2018 & 2019), showed an average of 3.49% grain yield improvement, by as much as 5.4% in a given season and up to 9.4% at certain trial locations. A subset of field trial locations was used to test for modification of ear traits and ФPSII, a proxy for photosynthesis. Results suggested that yield gain in transgenics could be associated with increased ФPSII, and the production of longer, thinner ears with more kernels. *ictB* localized primarily to the microsome fraction of leaf bundle-sheath cells, but not to chloroplasts. Extramembrane domains of *ictB* interacted *in vitro* with proteins involved in photosynthesis and carbohydrate metabolism. To our knowledge, this is the first published evidence of *ictB* insertion into a species using C_4_ photosynthesis and the largest-scale demonstration of grain yield enhancement from *ictB* insertion *in planta*. Results show that *ictB* is a valuable yield gene in the economically important crop maize, and are an important proof of concept that transgenic manipulation of photosynthesis can be used to create economically viable crop improvement traits.

## Introduction

Maize (*Zea mays* subs. *mays*) is the most widely grown cereal crop in the world with 194M ha planted worldwide in 2018, and the United States is the largest grower of maize accounting for 34% of the world’s production in 2018 (392M tonnes) [1]. While maize yields around the world have steadily increased over the past several decades, the rate of gain is not keeping pace with the needs of a growing population and new means of yield growth are needed [2]. Photosynthesis, the central process driving plant growth and yield, is an avenue with substantial potential for continued yield improvement [3–6]. Transgenic improvement of photosynthesis has led recently to increased biomass production in field trials of C_3_ crops [7] such as tobacco (*Nicotiana tabacum*) [8,9] and rice (*Oryza sativa*) [10]. Unlike these species, maize uses C_4_ photosynthesis, as do two other economically important crops, sugarcane (*Saccharum officinarum*) and sorghum (*Sorghum bicolor*), and multiple candidate bioenergy grasses such as miscanthus (*Miscanthus*) and switchgrass (*Panicum virgatum*). The unique biochemistry of C_4_ photosynthesis provides several opportunities for improvement [11].

C_3_ photosynthesis is the most common form of carbon assimilation in higher plants [12]. Atmospheric CO_2_ enters the plant leaf through stomata where it diffuses to chloroplasts in the mesophyll and is fixed into sugars via Rubisco and the Calvin-Benson Cycle. However, Rubisco is a relatively inefficient enzyme that can accept either CO_2_ or O_2_ at its active site, expending energy and generating a waste product when the latter is fixed. This process, i.e. photorespiration, is generally considered wasteful because it diverts energy away from photosynthesis. To overcome this inefficiency, C_4_ photosynthesis evolved by implementing a CO_2_ concentrating mechanism (CCM) that raises the CO_2_ concentration at Rubisco and therefore minimizes photorespiration [13]. In higher plants, C_4_ leaves typically develop the Kranz anatomy: numerous, enlarged bundle-sheath cells that serve as a largely gas-tight container for Rubisco, and a biochemical CCM which increases CO_2_ up to high concentrations in these cells [14]. This suppression of photorespiration typically leads to enhanced photosynthesis and water/ nutrient-use efficiency in C_4_ plants relative to C_3_ [3].

One ongoing effort to improve C_3_ photosynthesis is to engineer a CCM into C_3_ crops, effectively converting them to contain a C_4_-like photosynthetic apparatus [3,7,15]. Conversion to C_4_ requires a suite of metabolic and anatomical changes, whereas a potentially simpler option is to introduce carbon transporters found in cyanobacteria to actively concentrate CO_2_ around Rubisco [16–18]. Similar to C_4_ plants, cyanobacteria employ a diverse set of CCMs to overcome the challenges of performing photosynthesis across ranges of environmental conditions, and use many types of carbon transporters [19,20]. CCMs found in cyanobacteria often are simpler and controlled by fewer genes than those used by C_4_ plants, and therefore, are promising candidates for transgenic improvement of photosynthesis [16,21]. However, despite progress in the establishment of cyanobacterial CCM components in plants, improvements to yield in field conditions of the engineered plants have not yet been achieved [22–28].

The cyanobacterial membrane protein *ictB* was initially identified as a likely bicarbonate transporter, as mutants for the gene in *Synechococcus* sp. strain PCC 7942 had a high-CO_2_ requiring phenotype and impaired bicarbonate transport [29]. On this basis, *ictB* has been introduced into C_3_ plants in the hope that it would function as a CCM *in planta*. This has resulted in consistent increases in photosynthetic CO_2_ assimilation and often biomass and yield in arabidopsis (*Arabidopsis thaliana*), tobacco [30,31], rice [32,33], and soybean (*Glycine max*) [34].

Several studies have challenged the initial assumption that *ictB* functions as a bicarbonate transporter. Cyanobacteria mutants expressing endogenous *ictB* but with knockouts in five known C-uptake systems had a high CO_2_-requiring phenotype with no residual CO_2_ or bicarbonate uptake [35], suggesting *ictB* is not a direct bicarbonate transporter [16]. Another study in cyanobacteria tested for a possible indirect effect of *ictB* on bicarbonate transport, but showed that *ictB* did not induce a high affinity NA^+^ dependent bicarbonate transporter [36]. When compared with other cyanobacterial genes, *ictB* was identified as putatively involved in cross-membrane polymer export, with domains associated with oligosaccharide polymerization, rather than bicarbonate transport [37]. This suggests *ictB* does not serve as a functional CCM, and its enhancement of photosynthesis *in planta* stems from some different functionality. If so, *ictB* may be able to improve photosynthesis in species with an endogenous CCM, i.e. C_4_ plants, leading to enhanced carbohydrate production, and ultimately yield.

In this study, we tested the hypothesis that *ictB* insertion into the C_4_ crop maize would result in increased photosynthesis, carbohydrate production, and yield. We demonstrate that *ictB* insertion into maize does indeed enhance photosynthesis, carbohydrate production and yield, with improvements observed across multiple growing environments, years, locations, and germplasm. We demonstrate that *ictB* localized primarily to the microsome fraction of leaf bundle-sheath cells, but not to chloroplasts. We explore *in vitro* interactions of extramembrane domains of *ictB* with proteins involved in photosynthesis and carbohydrate metabolism, and discuss the implications for *ictB* function *in planta*.

## Materials and methods

### Generation of transgenic plants

Plasmid 130276 was generated as a single T-DNA containing the *ictB* and Phosphinothricin acetyltransferase (PAT) expression cassette. The sequence (US6320101 patent) was from *Synechococcus PCC 7942* encoding *ictB* protein. The *ictB* expression cassette was regulated by the *Zea mays* RbcS promoter, the 3′ untranslated region of the RbcS transcript and a nos terminator. The PAT gene from *Streptomyces viridochromogenes* [38,39] was regulated by the *Zea mays* ubiquitin promoter and the CaMV 35S terminator and was used as a selectable marker. The construct was verified by sequencing, and introduced into electrocompetent cells of *Agrobacterium tumefaciens* LBA4404 containing the superbinary vector pSB1 [40] to form superbinary plasmid pSB111-130276, which was verified by sequencing, for transformation.

Agrobacterium-mediated transformation of immature embryos of line B104 was used to generate transgenic events [41]. Standard tissue culture regime for bialaphos selection was used for generation of rooted transgenic plants. Transformed T0 plantlets were advanced to the greenhouse for soil acclimation and hardening. A single-copy insertion event (BHB1356) was selected, self-pollinated, and advanced for further evaluation. The resulting T1 plants that were homozygous for the insertion event were maintained as the positive event and null segregates of the insertion were maintained as the control.

### Growth chamber and greenhouse trials

To assess the effect of *ictB* on leaf carbohydrate production, inbred BHB1356 in the B104 background was grown in three consecutive experiments at the Donald Danforth Plant Science Center (St. Louis, Missouri, USA). The first experiment was conducted in a growth chamber (model BDW80, Conviron, Winnepeg, Canada) and the following two were conducted in a greenhouse. 5–8 plants of BHB1356 were grown in a randomized block design along with a null segregant used as a control.

Carbohydrate measurements were taken at 7 developmental stages across the three experiments: V5, V7, V9, V10, R1, R3, and R5 [42]. During vegetative growth, the youngest fully expanded leaf, as evidenced by ligule emergence, was sampled. During reproductive growth, the leaf extending from the developing ear was sampled. At each developmental timepoint, a small leaf punch was sampled at 5 timepoints: 10:00, 12:00, 15:00, 19:00, and 06:30 the following morning. Leaf discs were plunged into liquid N immediately after sampling and stored at -80°C until processing.

Foliar contents of carbohydrates were extracted in 80% and 50% (v/v) buffered (mM HEPES, pH 7.8) ethanol at 80°C. 5 consecutive incubations at 20 minutes each were performed to fully extract soluble carbohydrates [43]. Glucose, fructose, and sucrose were then measured using a continuous enzymatic assay following the protocol of [44]. For starch determination, the pellets of the ethanol extraction were solubilized by heating to 95°C in 0.1M NaOH and then acidified to pH 4.9 by adding a 0.5M HCl/0.1M sodium acetate solution [43]. Starch content was then determined by measuring glucose equivalents in the sample [45].

Glucose, fructose, sucrose, and starch were calculated as glucose equivalents using a glucose standard curve. The hexose pool was calculated by adding the glucose and fructose values together and the ratio of starch to total carbohydrates was calculated by dividing the starch measurements by the sum of the hexose, sucrose, and starch values.

### Field trials

#### Field trial setup

BHB1356 was tested for its hybrid performance across four growing seasons in North and South America. North American trials were conducted in maize-growing regions of the Midwest in the summers of 2018 and 2019 (2018S and 2019S, respectfully), while South American trials were conducted in maize-growing regions of Argentina in the winters of 2018 and 2019 (2018W and 2019W, respectively). Each plot consisted of two rows, 5.2–6.1 m long and spaced 0.76 m apart, with planting density of 7.4 plants m^-2^. Plots of BHB1356 were paired in-field with an adjacent control plot. Fertilization and irrigation were applied in line with regional agronomic practices at each site.

Details of variations in experimental setup (e.g. field trial locations, replication) across seasons and locations are given in Table 1 and S1 Table. A quality control process was implemented to remove low-quality data, which eliminated 27%, 4%, 12%, and 1% of plots in 2018S, 2018W, 2019S and 2019W, respectively (Table 1). Plots were defined as low quality if grain moisture content was not within 7–45%. Within each set of paired plots, data were only used if both plots of BHB1356 and control were of high quality, e.g. if the control plot was low quality then its paired BHB1356 plot was not used. Within each experimental block, control plots were used for further quality control. If the average block yield of these control plots was <100 kg ha^-1^, or had a coefficient of variation (CV) >0.2, then the entire block was considered to be of low quality. If a given plot’s stand density was not within 3 standard deviations of control plots in the same block, that plot was considered to be of low quality.

**Table 1:** Summary of experimental trials.

Season	N. of testers	ФPSII		Ear traits		Yield		
		N. of locations	Total plots used	N. of locations	Total plots used	N. of locations	Removed plots	Total plots used
2018S	2	2	22	2	7	10	11	35
2018W	4	0	0	6	69	6	3	69
2019S	7	4	67	7	141	16	41	295
2019W	10	0	0	4	118	4	1	179

The stable, inbred line homozygous for BHB1356 was crossed with up to ten male testers to generate hybrids containing a single copy of BHB1356 for field testing (Table 1). Null segregant hybrids were generated using the same method and were used as a control to test efficacy of BHB1356. Each location was arranged in a randomized split block design [46] to minimize field variation effects on transgene performance. Grain yield was measured at the end of the growing season at harvest and normalized to a grain moisture content of 15.5%.

#### Chlorophyll fluorescence measurements

As a proxy for photosynthesis, midday measurements of chlorophyll fluorescence were conducted during the US summer 2018–2019 growing seasons during vegetative and reproductive development in order to calculate the operating efficiency of photosystem II (ΦPSII). Chlorophyll fluorescence was measured using handheld fluorometers (FluorPen 110/S, Photon Systems Instruments, Drasov, Czech Republic). All measurements were taken on sunlit portions of mature leaves at the top of the canopy and three to five leaves from different plants were measured within each plot. ΦPSII was measured in three to five replicate plots of BHB1356 and control at each of a subset of field site locations (Table 1).

#### Ear traits

Five ears were sampled from the center of each plot from a subset of locations (Table 1). Ears were harvested at maturity and imaged using a MVEspigas imaging camera and corresponding MVInspector software (MachVision, Buenos Aires, Argentina). The images produced kernel number, ear length, ear width, and percentage of ear area with kernels from each imaged ear. In 2019S, ears were shelled using a Maizer SES single-ear sheller (ALMACO^®^, Nevada, Iowa 50201), pooled together, and subsampled to measure the weight of 100 kernels after adjusting to 15.5% grain moisture.

#### Selection of lead event BHB1356

Although several insertion events were created for *ictB*, this study focuses on characterization of the lead event BHB1356. This is based on testing of three events in a greenhouse experiment for leaf carbohydrate production (S1 Fig) and testing of ten events in field yield trials during three growing seasons (S2 Fig). Of the additional *ictB* events tested in both experiments, most events tended to outperform the null transgenic control both in starch and sucrose production at the end of the day (S1 Fig) and in grain yield (S2 Fig), and BHB1356 was selected as the lead event based on its superior performance across experiments.

### Tissue expression assays

#### Gene expression analyses by qRT-PCR

Samples from two independent biological replicates of greenhouse-grown inbred BHB1356 and negative control WT leaf (V6, R1, R4 and R6), pollen (R1), root (V6, R1, R4 and R6) and kernel (R6) from different development stages, were snap-frozen in liquid N2 and stored at -80°C. Total RNA was extracted using RNeasy plant mini kit followed by DNase I treatment (Qiagen). cDNA was synthesized from 1 μg of total RNA using M-MuLV Reverse Transcriptase (NEB) with Oligo-dT 23 VN (IDT) as primer. The resulting cDNA was used as template for qRT-PCR amplification in QuantStudio 6 Flex Real-Time PCR Systems (ThermoFisher Scientific) with PowerUP SYBR Green Master Mix (ThermoFisher Scientific). Three technical replicates were performed for each sample, in addition to negative controls without reverse transcriptase. The amplification program began at 95°C for 10 minutes, followed by 40 cycles of 95°C for 15 s and 60°C for 1 minute. Fluorescent signals were collected at each polymerization step. Expression was calculated as 2^-ΔΔCt^ [47] and normalized to that of the control gene ADH (alcohol dehydrogenase). Primers were designed to generate fragments between 80 and 150 bp using Primer3Plus [48]. *ictB* was amplified using primers, 13477, gggtggataggaattctgtgg and 13478, gttgtaggcacgaggtaagca. ADH gene was amplified using primers 12713, caggtggggtattcttggtg and 12714, atgttcgggtggaaaacctt.

#### Total protein extraction and Western-blotting

The same plant tissues used for RNA relative expression were also used for Western-blot analysis of *ictB*. *ictB* expression in various tissues was relatively low, therefore in order to improve detection resolution, *ictB* was enriched by immunoprecipitation for Western-blot. Total protein was extracted from100 mg of ground plant material in 1ml of extraction buffer (50 mM Tris, p H 7.5, 150 mM NaCl, 0.8% DDM, 0.2% Tween) using bead-ruptor 12 homogenizer (speed at 3.1, time for 2 minutes). Cell lysate were precleared with agarose resin (Pierce, Cat # 26150) for 30 minutes, then incubated with 5 μg of anti-BH15-1 antibody for 1 h at 4°C. Protein A/G agarose resin (Pierce, Cat# 20422) was added to reaction, then incubated additional 1 h at 4°C. The resin was washed three times with EB buffer for each 5 minutes. Enriched protein was eluted from resin with 2x SDS buffer and heat treated at 60°C for 20 minutes. The eluted *ictB* protein was analyzed by Western-blot using an antibody developed to react to the *ictB* C-terminus. BH15-1 antibody was generated by GenScript (Piscataway, New Jersey, USA) in use for immunoprecipitation. A polyclonal antibody directed against N-terminal *ictB* protein was produced in rabbits and named as BH15-1.

### Subcellular localization

#### Confocal microscopy

A Leica TCS SP2 confocal laser scanning inverted microscope (Leica Microsystemes, Wetzlar, Germany) equipped with 63X water immersion objective was used to examine the subcellular localization of *ictB-GFP* in leaf cross-sections. To study the localization of *ictB*, a construct of *ictB* fused with GFP tag at C terminal under RbcS promoter was used for confocal analysis. *ictB-GFP* construct was transformed into maize by stable transformation. At least three independent lines with different copy number were tested for GFP localization and observed consistent results. To visualize GFP fluorescence, excitation with a 488 nm argon laser was used and emission was detected at between 500–530 nm. Collected images were analyzed using Leica Application Suites -X (Leica Microsystems, Germany).

#### Immunolocalization assays

Chloroplast purification was performed followed by Chloroplast Isolation Kit (Sigma, CP-ISO). Total protein from intact chloroplast was solubilized in 1x chloroplast isolation buffer with 0.8% DDM. Microsome isolation [49] and mesophyll and bundle sheath cell isolation [50] were performed with ground leaf tissue powder.

Presence of *ictB* in mesophyll and bundle-sheath cell leaf fractions, and in cytosol, microsome, and chloroplast protein fractions, was tested by Western-blot gels using an antibody developed to react to the *ictB* C-terminus. Tic 40 antibody (Agrisera, AS10 709) was used as a chloroplast marker.

#### *In-Planta* T-DNA sequence junction determination

The *in-planta* sequence of the T-DNA was obtained through multiple waves of NGS sequencing including sequence pull-downs, as well as whole genome sequencing resulting in >500X coverage. Illumina short-read sequencing, PacBio and Oxford Nanopore long-read sequencing were employed to identify the junction sequence. Sequences were aligned to the B73 reference genome (version B73 RefGen_v4, AGPv4, NCBI Assembly ID: GCF_000005005.2). High confidence *in-planta* sequence of the T-DNA was subsequently obtained through NGS based analysis of overlapping cloned PCR products of the T-DNA locus. These PCR products were cloned into TOPO vectors and multiple clones for each segment were sequenced using NGS. This led to high confidence sequence of the T-DNA insertion in BHB1356 and at least 2kb of flanking sequence of the insertion locus.

### Protein-protein interaction assays

The *ictB* protein was analyzed by Protter (http://wlab.ethz.ch/protter/start/), a predictive algorithm that visualizes the sequence, topology and annotations of individual proteins (S3 Fig). Because *ictB* is an integrated membrane protein with hydrophobic and hydrophilic components, attempts to synthesize it in its entirety outside of a membrane resulted in loss of 3D structure that would have rendered protein-protein interaction assays meaningless. Instead, three of the protein’s extramembrane domains were synthesized and tested: C-terminus, N-terminus, and Big Loop Region (S3 Fig) for yeast two hybrid screening and 1:1 interaction to validate the screening results. Interaction of *ictB* protein with other plant proteins was then tested *in vitro* via several assays.

Yeast two-hybrid screening was performed by Hybrigenics Services, S.A.S., Evry, France as in [51], using the LexA/Gal4 binary transcriptional system to test the interaction of *ictB* with a prey library of *Z*. *mays* leaves and ovaries. A subset of 18 proteins were selected with moderate to very high confidence interaction with *ictB*, and whose annotated function suggested a possible role in enhancing photosynthesis and yield (S2 Table). These were then used in validation yeast two-hybrid assays testing 1:1 interaction with *ictB* [51].

A subset of 4 proteins was tested to further validate 1:1 interaction with either full length or a fragment of *ictB* protein (229–467 aa, which contains big loop and C-terminus regions). These 4 proteins, along with *ictB*, were recombined with pET 52b and pET_28a vectors respectively to produce His-tagged fusion protein, MBP, or Flag fusion proteins. Expression of recombinant protein was induced at 16°C for overnight with 0.2 m M IPTG. Fusion protein was extracted by lysis buffer (25 mM Tris pH 7.0, 0.5M NaCl, 10% glycerol, 1% protease inhibitor cocktail) and purified using NTA resin (Qiagen) or MBP resin (New England). *In vitro* pull- down assay was performed to confirm 1:1 interaction with *ictB* [52].

### Statistical analysis

#### Growth chamber and greenhouse trials

Measurements of leaf carbohydrate contents were recorded repeatedly from plants to capture data from multiple developmental stages. Therefore, a repeated measures linear mixed model was developed for each measured time of day (10:00, 12:00, 15:00, 19:00, and 6:30). Fixed effects in the model included development stage and *ictB*/control, and their interaction. Carbohydrate contents were determined in multiple assays, therefore a random intercept was included for each assay to account for variability between analyses. A random intercept also was included for each plant to address repeated observations, and the best-fit autocorrelation structure was selected via Akaike Information Criterion (AIC) [53]. After the repeated measures model was fit, one-tailed, two-sample *t*-tests were performed to evaluate whether carbohydrate content of transgenics was greater than control. Statistical analysis was performed in R v3.6.2 [54] using the nlme package [55]. We considered difference in this test and throughout the study to be statistically significant at α = 0.05. Across all analyses, histograms of residuals were visually examined to ensure that the assumption of normality was not violated.

#### Field trials

For traits measured in the field, differences between BHB1356 and control were based on paired plots to remove the influence of in-field environmental effects. The evaluation measure for each trait was the difference (Δ) between transgenic and control values, in the same paired plot and the same tester. For yield and ФPSII, a one-tailed, one-sample *t*-test was then used to assess whether Δ was greater than 0, for each measured trait and each growing season (2018S, 2018W, 2019S, 2019W). For all other field traits, a two-tailed, one-sample *t*-test was used to test whether Δ differed from 0 [56]. Δ was assumed to have homogeneous variances within a trial. Statistical analysis was performed in base R v3.6.2 [54].

## Results

### Growth chamber and greenhouse trials

**Fig 1:**
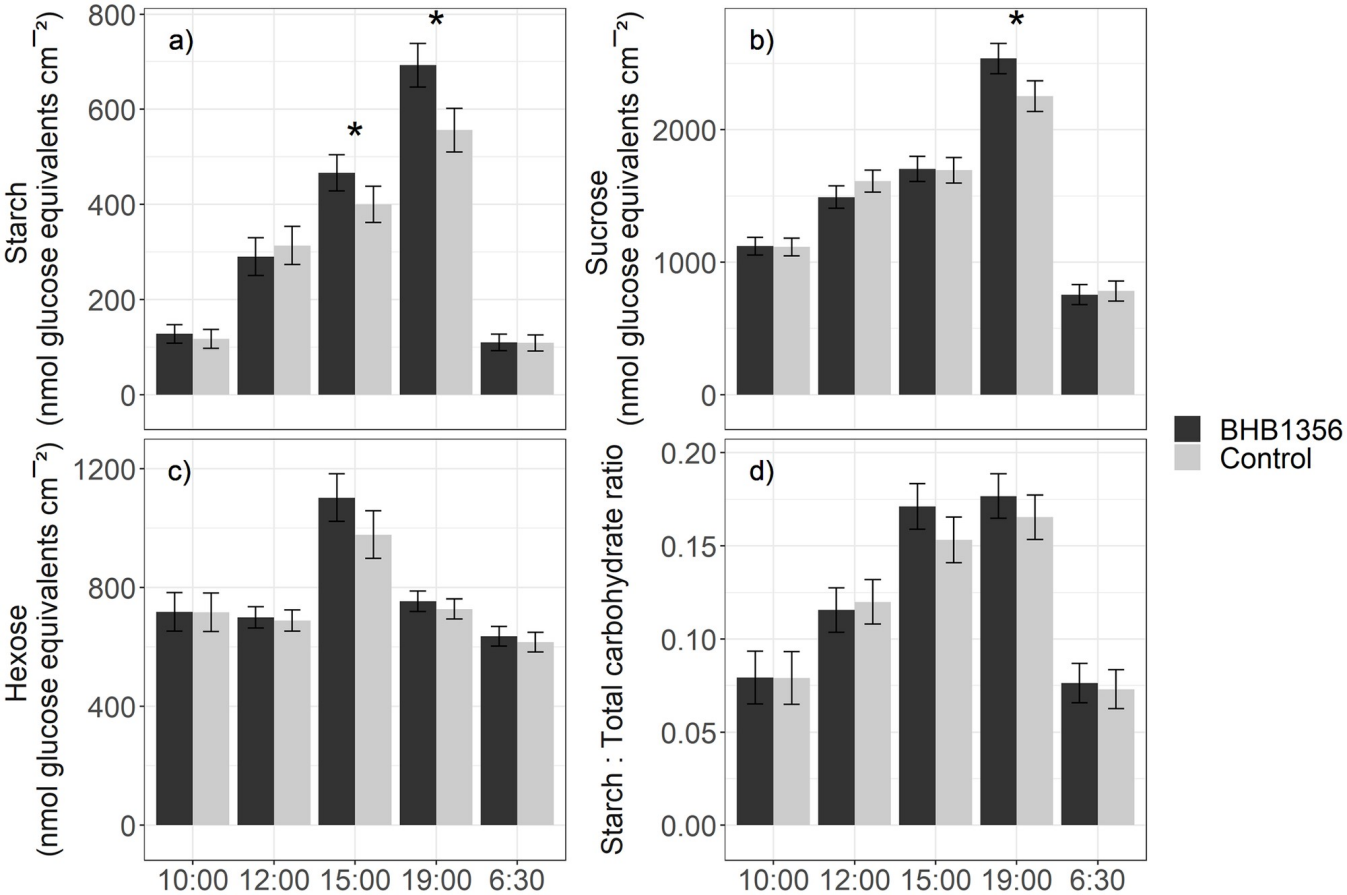
Leaf carbohydrate content measured at different timepoints in controlled-environment experiments. a) starch, b) sucrose, c) hexose, d) starch:total carbohydrate ratio. Bars show the LSmean, error bars give the LSD05/2. * indicates significant difference (*P*<0.05) from a one-tailed two-sample t-test testing BHB1356>control.

Starch and sucrose content increased throughout the day, and were at their lowest at the end of the night (6:30 timepoint, Fig 1a and 1b). Across vegetative and reproductive stages, leaf starch content was greater in BHB1356 inbreds than control at the 15:00 (17%, *P* = 0.045, *t* = 1.71, Fig 1a) and 19:00 timepoints (25%, *P* = 0.002, *t* = 2.95, Fig 1a). Similarly, leaf sucrose content was greater in BHB1356 inbreds than control at the 19:00 timepoint (13%, *P* = 0.008, *t* = 2.46, Fig 1b). There was no difference in starch and sucrose content between BHB1356 and control at other timepoints, including the 6:30 timepoint at the end of the night. There were no differences between BHB1356 and control for leaf hexose content or for starch:total carbohydrate ratio at any timepoint (Fig 1c and 1d).

### Field trials

**Fig 2:**
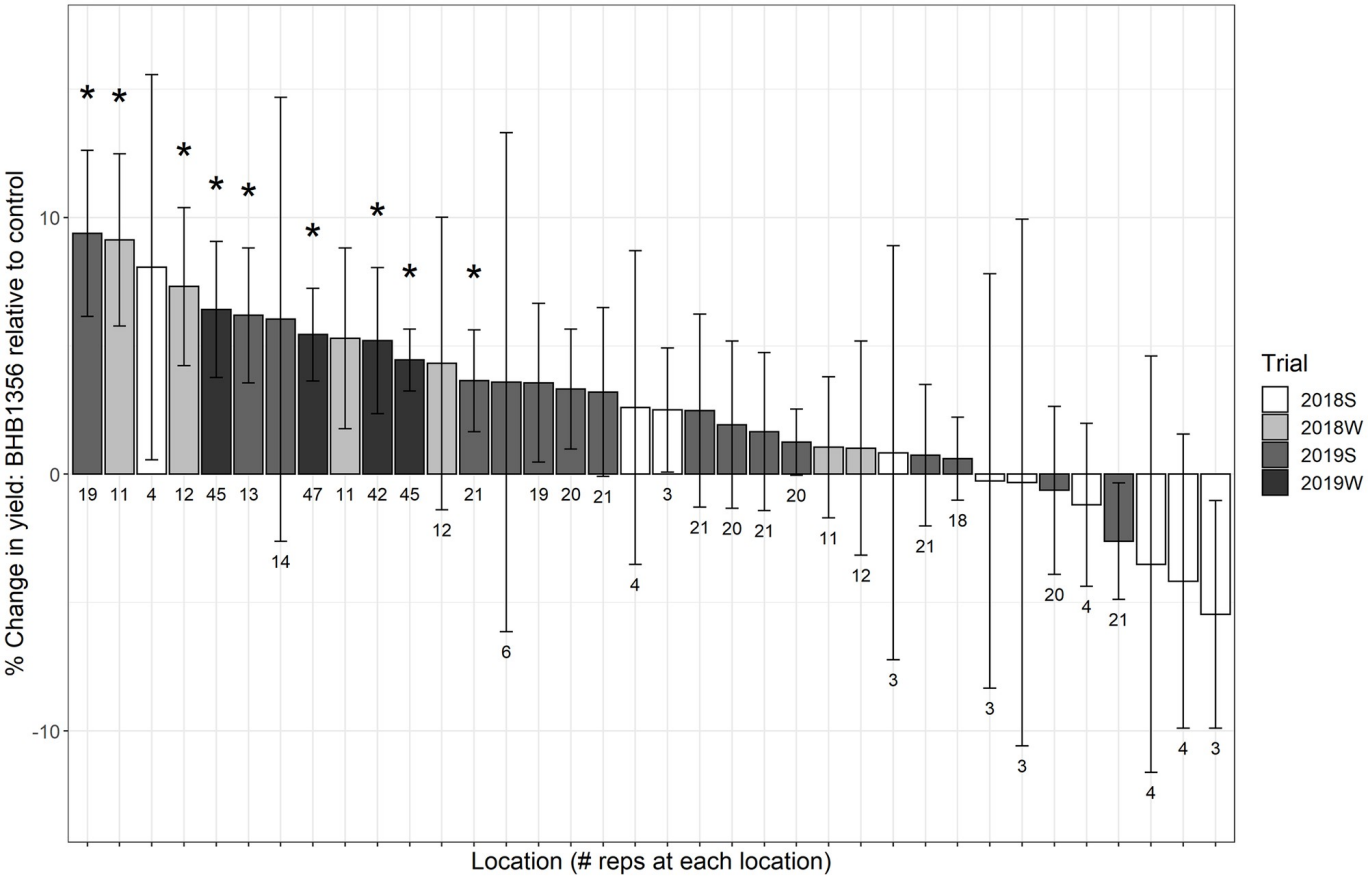
% difference between BHB1356 and control for yield, grouped by location, in the four growing seasons: 2018S, 2018W, 2019S, 2019W. Bars are mean ± standard error. * indicates significant difference (*P*<0.05) of the Δ (i.e. BHB1356 minus control) from 0 based on a two-tailed *t*-test.

**Fig 3:**
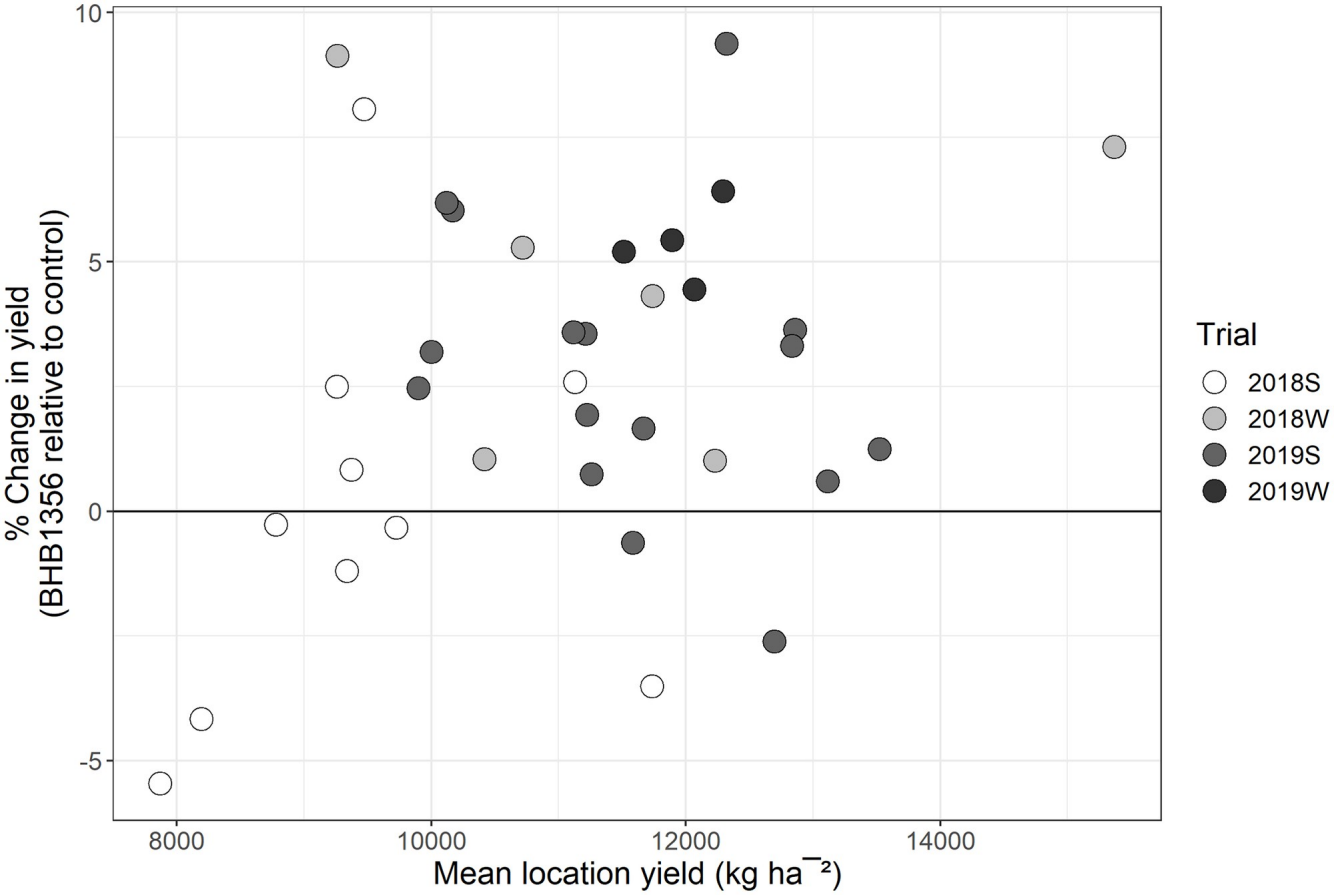
% Change in yield for BHB1356 relative to control in multiple field trials, grouped by location, and plotted against mean location yield.

In highly replicated trials spanning three growing seasons, BHB1356 had an overall yield increase of 3.49% relative to control (*P*<0.001, *t* = 6.09, Table 2). Despite improved yields in 2019W (5.4%, *P*<0.001, *t* = 4.97, S2 Table), 2018W (4.7%, *P* = 0.002, *t* = 2.91, S2 Table) and 2019S (2.4%, *P* = 0.001, *t* = 3.11, S2 Table), there was no trial-wide yield increase in 2018S. When assessing BHB1356 performance at each growing location independently, there was up to a 9.4% increase in yield of BHB1356 over control with 78% of locations trending positive in terms of performance over control (Fig 2). 2018S had considerably lower sample size per location which led to greater variation within those results when looking at locations independently. Further, 2018S represented the majority of locations that trended negative for performance. There was no correlation between effect size of yield increases of BHB1356 at a given location and absolute yield performance at that location (*P* = 0.197, Fig 3), suggesting the yield advantage of BHB1356 was not conditional to low- or high-yielding environments. The increased yield of transgenic plants was not accompanied with deleterious agronomic side-effects such as increased root or stalk lodging, or decreased stand count or plant height (S4 Fig).

**Table 2:** Mean values for traits measured in field experiments.

Trait	BHB1356	Control	Δ	% Change	*t* statistic	*P*
Yield (kg ha^-^¹)	11448.3	11062.3	386.0	3.5	6.09	**<0.001**
Kernel number	505	491	14	2.9	5.01	**<0.001**
Ear width (cm)	53.8	54.1	-0.3	-0.6	-2.52	**0.012**
Ear length (cm)	180.4	177.5	2.9	1.6	4.32	**<0.001**
Ear area with kernels (cm^2^)	94.2	94.2	0.0	0.0	-0.36	0.719
100 seed weight (g)	33.3	34.0	-0.7	-2.1	-1.65	0.103
ФPSII, vegetative stage (mol mol^-^¹)	0.42	0.41	0.01	1.7	1.20	0.119
ФPSII, reproductive stage (mol mol^-^¹)	0.45	0.43	0.02	4.8	2.90	**0.002**

Δ gives the difference between BHB1356 and control, and the associated % change is also shown. *P*-values are the result of a two-tailed *t*-test evaluating Δ≠0 for all ear traits, and a one-tailed *t*-test evaluating Δ>0 for yield and ФPSII. 100 seed weight was measured in 2019S, ФPSII was measured in 2018S and 2019S, and all other traits were measured in 2018S, 2018W, 2019S, and 2019W.

Overall, ФPSII in BHB1356 hybrids increased relative to control in reproductive stage leaves (4.8%, *P* = 0.002, *t* = 2.9, Table 2), but not in vegetative stage leaves. When results were split by growing season, ФPSII in BHB1356 hybrids increased relative to control in vegetative stage leaves in 2019S (2.9%, *P* = 0.036, *t* = 1.83, S2 Table), but not in 2018S, and reproductive stage leaves in 2018S (15.5%, *P* = 0.002, *t* = 3.27, S2 Table), but not in 2019S (S2 Table).

Overall, ears of BHB1356 hybrids had increased kernel numbers (2.94%, *P*<0.001, *t* = 5.01, Table 2) on longer (1.62%, *P*<0.001, *t* = 4.32, Table 2), thinner (-0.49%, *P* = 0.012, *t* = -2.52, Table 2) ears. Kernel number trended positive across all four growing seasons, but increases were only significant in 2019S (3.8%, *P*<0.001, *t* = 3.8, S2 Table) and 2019W (2.3%, *P* = .011, *t* = 2.6, S2 Table). There was an increase in ear length in BHB1356 hybrids relative to the control in 2018W (2.6%, *P* = 0.004, *t* = 3, S2 Table) and 2019S (1.7%, *P* = 0.008, *t* = 2.7, S2 Table) but not in 2018S or 2019W. Similarly, there was a decrease in ear width in BHB1356 hybrids relative to the control in 2018W (-1%, *P* = 0.014, *t* = -2.51, S2 Table) and 2019S (-0.9%, *P* = 0.013, *t* = -2.52, S2 Table) but not in 2018S or 2019W. There were no differences between BHB1356 and control for ear area with kernels or 100 seed weight (Table 2).

### *ictB* localizes to the microsome of leaf bundle-sheath cells, but not to chloroplasts

**Fig 4:**
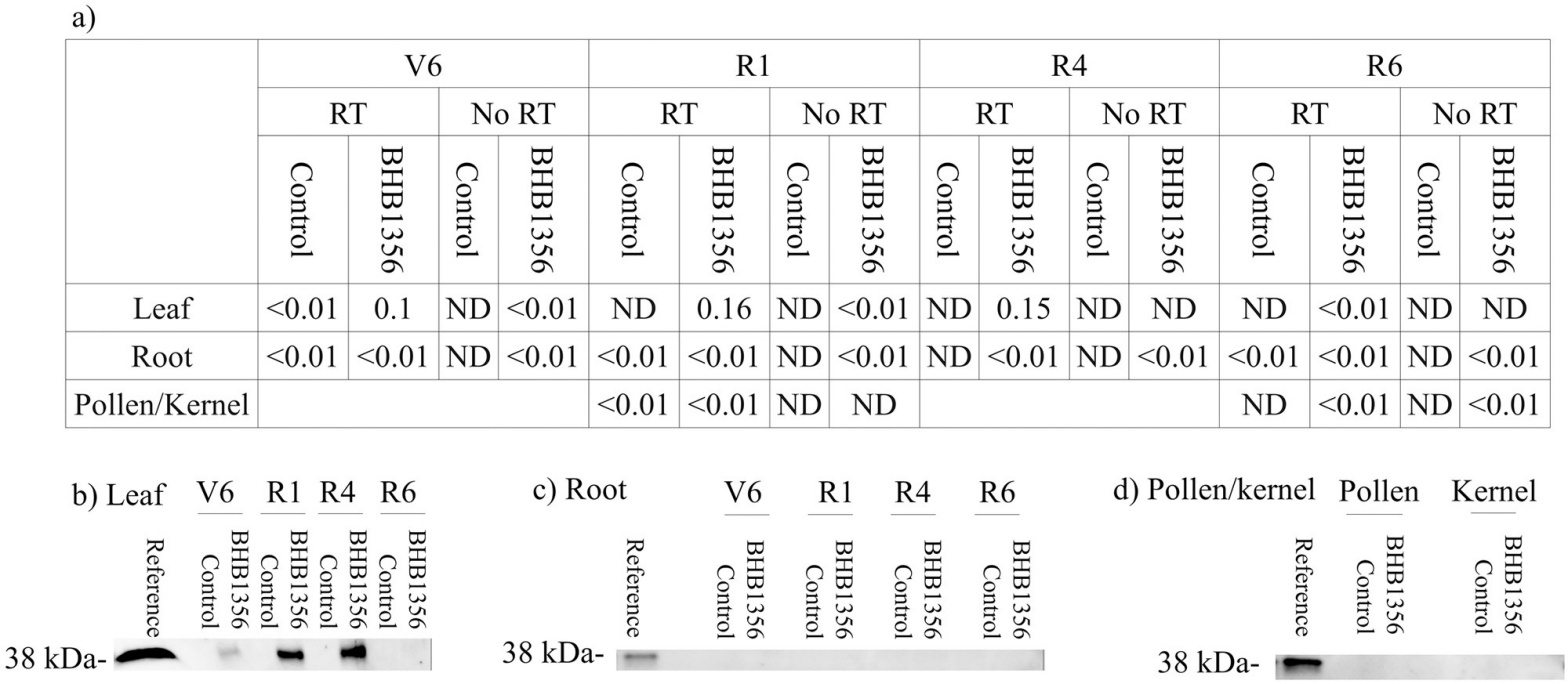
RNA and protein expression of *ictB* in different plant tissues. a) relative RNA expression of *ictB* gene, normalized to expression of actin, and b-d) Western-blots showing immunoprecipitation of *ictB* protein in transgenics and negative control WT plants. Samples of the purified *ictB* protein served as a reference. Pollen and kernels were sampled at R1 and R6, respectively. RT: reverse transcriptase; ND: not detected.

RNA expression (Fig 4a) and protein abundance (Fig 4b–4d) were only observed in leaves at three of the four developmental stages measured (V6, R1, and R4). In all other tissues, there was no detectable *ictB* protein, and RNA expression was <1% of the reference gene actin (Fig 4). *ictB* RNA expression was also <1% of the reference gene actin in assays without reverse transcriptase (Fig 4a).

**Fig 5:**
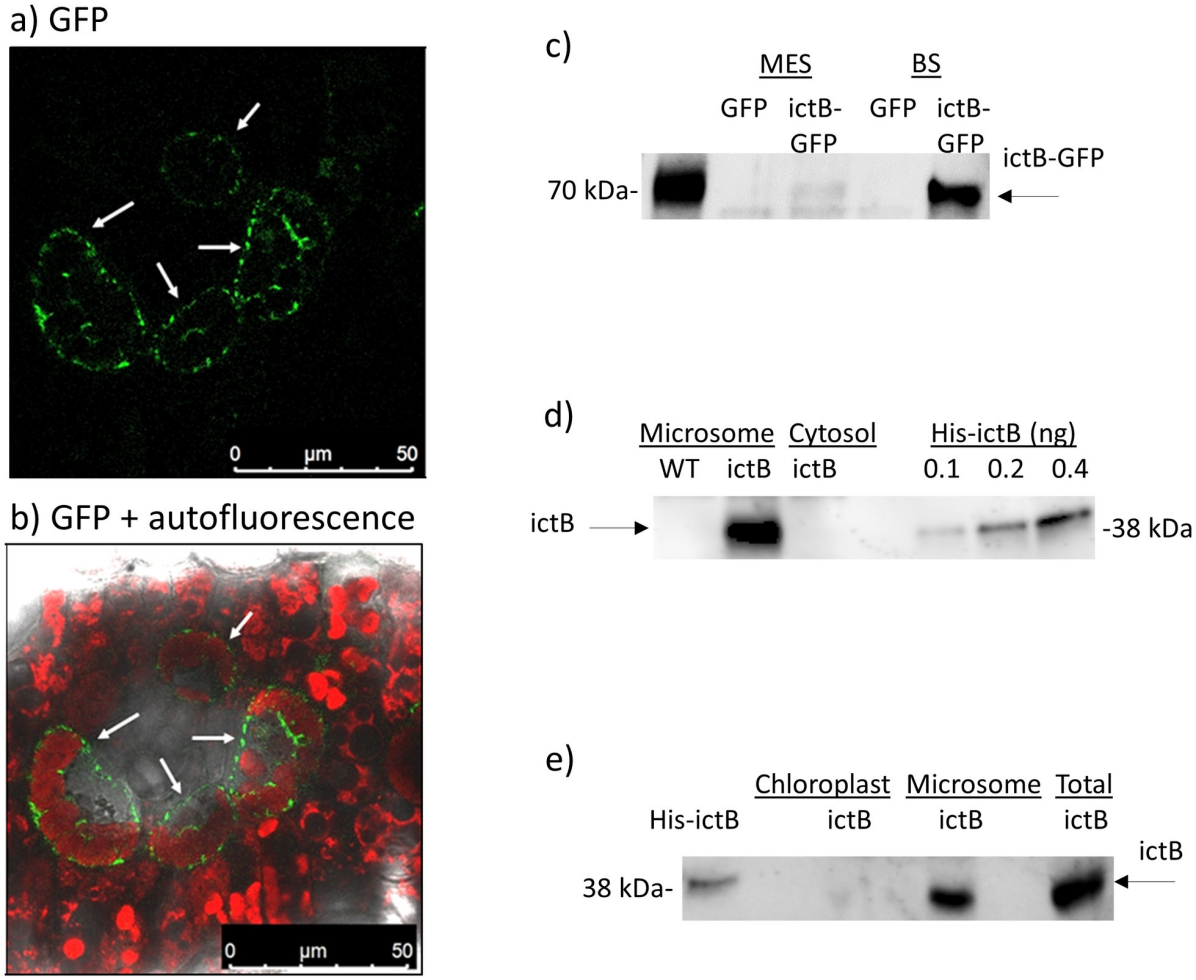
Subcellular localization of *ictB*. a) confocal images of leaf cross-sections of *Z*. *mays* transgenic event 1356, showing GFP signal from *ictB* protein. White arrows show bundle-sheath cells. 63X magnification. b) as in panel a), but merged with bright-field and chlorophyll autofluorescence. c) Western-blot of *ictB*-GFP and GFP protein in mesophyll (MES) and bundle-sheath (BS) cells. d) Western-blot of *ictB* protein in the microsome and cytosol protein fraction. Samples of His-*ictB* protein of various concentrations are included as a reference, and a WT plant sample is included as a negative control. e) Western-blot of *ictB* protein in chloroplast, microsome, and total protein fraction.

Analysis of fluorescence of GFP-tagged *ictB* in leaf cross-sections was observed primarily in bundle-sheath, not mesophyll cells (Fig 5a and 5b). GFP fluorescence did not appear to overlap with chloroplasts; suggesting *ictB* was not localized within chloroplasts. This was confirmed in Western-blots, with the bulk of *ictB* localized to bundle-sheath, not mesophyll cells (Fig 5c). Further, *ictB* was present in the microsome protein fraction, but not in the cytosol (Fig 5d). Within the microsome, *ictB* was absent from chloroplast membranes (Fig 5e).

### The BHB1356 event has a T-DNA insertion in chromosome 2

The BHB1356 locus was sequenced through multiple NGS platforms to a coverage of 30X and subsequently using an overlapping PCR product strategy, the 5’and 3’ flanking genomic sequences to the T-DNA insertion were sequenced at >8000X coverage to get high confidence sequence information. Based on cumulative data, the T-DNA insertion junction was identified as a unique location at chromosome 2, location NC_024460.2:197,383,549 on the B73 genome, at least 1.5kb away from any known genes.

### *ictB* domains interact with proteins involved in photosynthesis and carbohydrate metabolism

35 annotated proteins were found to interact with *ictB* domains with moderate to very high confidence (S3 Table). Of these, two (cl8519_1a and glu2) interacted with both N-terminus and Big Loop domains, which are found on opposite sides of the membrane (S3 Fig). 18 proteins were selected because their annotation suggested a possible function affecting yield, and all but one of these (LOC100216660, an Armadillo/beta-catenin-like repeat) were confirmed to have 1:1 interaction with *ictB* in validation Y2H assays (Table 3). Four of these (Molecular chaperone Hsp40/DnaJ family protein, beta-D-glucosidase precursor (glu2), Fructose-bisphosphate aldolase, phosphoenolpyruvate carboxylase 4) were selected and again confirmed to have 1:1 interaction with *ictB* in validation pull-down assays (S3 Table, S5 Fig).

**Table 3:** Proteins with *ictB* interaction confirmed in Y2H 1:1 assay.

General function	Protein name
C_3_ cycle + glycolysis	Fructose-bisphosphate aldolase
C_4_ cycle	NADP malic enzyme 3 (me3); Pyruvate orthophosphate dikinase 1 (pdk1); Phosphoenolpyruvate carboxylase 4; Malate dehydrogenase 5
Glycolysis	Enolase 2 (eno2); Cytosolic glyceraldehyde-3-phosphate dehydrogenase
Starch synthesis	ADP-glucose pyrophosphorylase
Glycine degradation	Glycine dehydrogenase (decarboxylating), mitochondrial
Protein interaction	Ankyrin repeat domain-containing protein 2; Signal recognition particle 54 kDa protein chloroplastic
Chaperonin	Molecular chaperone Hsp40/DnaJ family protein; Translocon Tic40
Stress response	Beta-glucosidase 44; beta-D-glucosidase precursor (glu2); Serine hydroxymethyltransferase

## Discussion

This study investigated whether the insertion of *ictB* into the C_4_ crop maize would lead to improved photosynthesis, carbohydrate production, and ultimately yield, as previous studies have demonstrated in C_3_ crops. To test this hypothesis, maize plants were transformed to contain the *ictB* transgene and tested in both field and controlled environments across multiple studies. Field trials across four growing seasons were used to assess agronomic performance along with photosynthetic performance in two growing seasons, whereas carbohydrate production was measured in three consecutive greenhouse and growth chamber experiments.

### *ictB* insertion boosts carbohydrate production in leaves

Carbohydrate production is a fundamental intermediate step between photosynthesis and yield, as carbon fixed through photosynthesis is converted into sucrose and transported to sink tissues for growth and grain fill, or converted into starch as temporary storage to support leaf metabolism at night [57,58]. The finding of increased starch and sucrose accumulation in leaves of *ictB* transgenics at the end of the day (Fig 1) is supported by a previous study in which *ictB* insertion increased water soluble carbohydrate production in field-grown rice [32]. Carbon partitioning into sucrose and starch is tightly regulated by feedback mechanisms that generate starch when sucrose production exceeds export, while sucrose synthesis at night results from degradation of starch in the bundle-sheath [58,59]. Leaf hexose content and starch:total carbohydrate ratio were not different between BHB1356 and control at any timepoint, suggesting this highly regulated system was not disrupted in BHB1356 (Fig 1).

Increased carbohydrate production can trigger a negative feedback loop, as cumulating sucrose within the leaf can result in down-regulation of photosynthetic activity. However, this is only expected to occur in plants that are sink-limited: conversely, in source-limited plants, increased carbohydrate synthesis should enable greater plant growth [60]. Starch content at the end of the night can be measured as a proxy indicating whether plants are source or sink-limited, where larger starch pools indicate sink-limitation, and small starch pools indicate source-limitation [61]. This is because allocating carbon into starch reserves is an energetically intensive process and so it is beneficial only to store as much starch as the plant needs to get through the night [62]. Here, at the end of the night (6:30 timepoint, Fig 1), starch reserves were at their lowest, and there was no difference in starch content between BHB1356 and control. This suggests that plants were source-limited, and that the extra starch accumulated at the end of the day in BHB1356 was used for plant growth and grain fill. In mature maize leaves, and during the day, sucrose and starch production are largely confined to the mesophyll and bundle-sheath, respectively [63]. This spatial separation may help prevent negative feedback of sucrose accumulation on bundle-sheath-localized Calvin cycle enzymes during the day, when photosynthesis is active [58].

### BHB1356 increases yield in the field

The increased performance of BHB1356 hybrids in field trials suggests that enhanced carbohydrate production translated to increased grain yield (Table 2). These yield increases appeared to be associated with improved ФPSII, suggesting that an improvement to photosynthesis was the basis for increased carbohydrate production and yield of BHB1356 hybrids (Table 2). However, the ФPSII advantage of BHB1356 was not seen as consistently as the yield advantage. This is contrary to past insertions of *ictB in planta*, which have improved photosynthesis more reliably than grain yield [30–34]. Several factors may underlie this apparent discrepancy. ФPSII measurements were taken at a subset of locations, some of which were relatively low-performance locations for BHB1356: for instance, one location in 2019S near Shipman, IL, had among the lowest values for Δ yield. In 2018S, this location was used for ФPSII measurement, but due to weather damage could not be used for yield measurement. Each location was also only measured once during vegetative and reproductive growth and does not fully represent photosynthetic performance across the entire growing season but rather a snapshot at measurement time. Finally, it should be noted that ФPSII is a proxy for photosynthesis [64]. Some important photosynthetic processes occur downstream of PSII, such as partitioning of energetic compounds (ATP) and reducing equivalents (NADPH) [64] which may have led to increased photosynthesis and yield, despite having less impact to ФPSII.

At the ear level, increased grain yield in BHB1356 hybrids resulted from increased kernel numbers on longer, thinner ears (Table 2). Kernel number is determined relatively early in the maize growth cycle, in the first 15 days after pollination [65], suggesting the effects of *ictB* insertion were already active at this time. Often, improvements in ear traits aligned with overall yield results for the given trial, but some discrepancies existed most likely due to ear traits being measured at a subsample of plants within a plot and locations for a given trial. For example, one of the largest discrepancies was between kernel number and yield in the 2018S trial, but this can be attributed to collecting ear trait data from only 2 of the 10 total locations that yield was measured in (Table 1).

### Variation in yield across locations and growing seasons

Despite a consistent trend of increased yield, there was considerable variation in the effect size of BHB1356 hybrids across locations and growing seasons (Fig 2). Although we found no evidence that the yield advantage of BHB1356 was conditional to low- or high-yielding environments (Fig 3), photosynthesis is a tightly regulated metabolic process that has many other layers of control, including environmental factors, that could impact the effect size of genetic improvements to photosynthesis and yield [66].

The trials conducted in this study were in geographically distinct regions of the United States and Argentina over multiple years, and therefore experienced different environmental conditions. Variation in external factors such as water availability or temperature across locations and growing seasons could affect a photosynthesis trait. A central feature of drought response in plants is a reduction of stomatal conductance to restrict water loss through transpiration [67–69], which in turn, can limit CO_2_ supply within the leaf and reduce photosynthesis. Because carbon uptake by the plant is tied to water use, water limitation could potentially reduce or even nullify the efficacy of a photosynthesis trait [4]. Variability in weather and soil characteristics across locations, including density, clay and organic matter content, can be expected to influence water availability for plants [70]. Photosynthesis and dark respiration of maize both increase with temperature, but above 30°C photosynthesis declines while dark respiration continues to increase [71,72]. At such high temperatures, the relative simulation of respiration could cut into the net carbon gain of a photosynthesis trait and mitigate benefits to productivity and yield [73].

Another potential factor contributing to variation among locations of BHB1356 performance was the planting date of each location. Planting dates in this study were up to 3 weeks apart and, in the Midwestern United States, were outside of the ideal planting window for maize which is typically April 15 to May 15. A summary of 6 studies measuring impact of planting time on the yield potential of maize demonstrated that late May and early June plantings of corn can have over 20% reduction in absolute yield [74]. Late plantings shorten the growing period and may reduce the efficacy of a photosynthetic trait as increased yields are theoretically the result of the cumulative effect of increased carbon fixation over time. Further, later plantings push critical developmental stages into periods of the growing season that tend to be less optimal for yield, such as pollination in high temperatures [74]. Although planting date effect on BHB1356 performance was not explicitly tested in this study, the 5 locations that had the highest effect size of BHB1356 in 2019S were among the 7 locations that were planted closer to the optimal planting window. Additional studies are needed to test whether the yield enhancement of BHB1356 may be increased and more consistent if planting occurs within the ideal planting window.

### *ictB* localizes to the bundle-sheath and interacts with photosynthetic proteins *in vitro*

Because of its potential role in enhancing photosynthesis, *ictB* expression was targeted to leaf tissue using the RbcS promoter, unlike prior studies which used a constitutive CaMV 35S promoter and so would expect expression throughout the plant [32,34]. Accordingly, *ictB* expression was demonstrated in leaves, and not in other tissues (Fig 4). *ictB* was not expressed in leaves at the R6 growth stage, possibly because the protein was associated with a Rubisco promoter, and Rubisco expression should decline at the end of the plant’s growing cycle when photosynthetic activity ceases.

*ictB* has been hypothesized to function as a bicarbonate transporter when inserted into C_3_ plants, enabling increased mesophyll conductance (*g*_*m*_), improved Rubisco access to CO_2_, and culminating in increased photosynthetic CO_2_ uptake [32,34]. In a C_4_ plant such as *Z*. *mays*, bicarbonate is fixed by phosphoenolpyruvate carboxylase (PEPc) in the mesophyll cytosol [11,75]. After this initial step, C delivery to Rubisco in the bundle-sheath is not ensured by bicarbonate, but by shuttling of metabolites including malate and aspartate via the C_4_ cycle [11,75]. Therefore, increased *g*_*m*_ should only benefit photosynthesis to the extent that it facilitates PEPc access to bicarbonate under low-CO_2_ conditions [76]. However, we found very little *ictB* in mesophyll cells of the transgenic plants studied here (Fig 5). Instead, *ictB* localized primarily to the microsome of bundle-sheath cells, where increased conductance to bicarbonate would not be expected to boost photosynthesis (Fig 5). To our knowledge, the only other existing documentation of *ictB* localization upon transformation into a higher plant is in the C_3_ grass rice, where it was reportedly expressed in the MES cytoplasm [32].

*ictB* was found to interact with several proteins that localize to the chloroplast [77,78] and/or are involved in photosynthesis (e.g. NADP malic enzyme (NADP-ME), pyruvate orthophosphate dikinase (PPDK), PEPc, malate dehydrogenase (MDH), Fructose-bisphosphate aldolase (FBPase), Table 3 and S3 Table). This supports the hypothesis that *ictB* enhances photosynthesis upon insertion *in planta*. The finding that *ictB* and FBPase interact is notable, as combined over-expression of *ictB* and FBPase, along with another C_3_ photosynthetic enzyme SBPase, has resulted in greater enhancement to photosynthesis in tobacco [31] and rice [32] than when *ictB* was expressed alone. However, *ictB* itself was not found in chloroplasts of the transgenic plants studied here (Fig 5), suggesting insertion into non-chloroplastic membranes such as endoplasmic reticulum or plasma membrane. Cytosol-oriented domains of *ictB* may have performed post-translational modifications to photosynthetic proteins to improve their performance prior to chloroplast insertion.

*ictB* was found to interact with enzymes involved in glycolysis (FBPase, enolase, cytosolic glyceraldehyde-3-phosphate dehydrogenase (GAPC2)) and starch synthesis (ADP-glucose pyrophosphorylase, Table 3 and S3 Table), which operate in the cell cytoplasm and so could have interacted with *ictB in planta* [63]. This suggests that the increased starch and sucrose content of transgenic maize plants studied here (Fig 1) may have resulted from *ictB* improving carbohydrate metabolism downstream of photosynthesis.

## Conclusion

Enhanced photosynthesis is a promising avenue to enhance crop yields, and *ictB* has proven potential for photosynthetic improvement. Still, transgenic improvements to photosynthesis have struggled to translate to improved grain yield in crops, although there have been recent successes in rice [79,80] and maize [81]. This may be because of negative feedback effects on photosynthesis, or external abiotic factors such as water limitation that restrict yield enhancements to specific environments and growing conditions [4]. Here we show the first published incidence of *ictB* insertion into a plant species using C_4_ photosynthesis, and the largest-scale demonstration of grain yield enhancement from *ictB* insertion *in planta*. While future research is needed to fully understand the magnitude of the effect *ictB* has on yield, these results are a promising step towards increasing grain yield of staple crops such as maize. Together with results of *ictB* transgenics in C_3_ species [30–34], the results here in maize suggest that *ictB* is a remarkably broad-spectrum yield gene, able to improve plant growth across various taxonomic groups and photosynthetic types.

## Supporting information

S1 Fig Leaf carbohydrate content measured at different timepoints in a controlled-environment experiment with 3 transgenic *ictB* insertion events including the lead event 1356. (DOCX)

S2 Fig Difference between transgenic and control (Δ) for grain yield across multiple field trials, testers and locations, in 10 insertion events including the lead event 1356. (DOCX)

S3 Fig *ictB* structure. (DOCX)

S4 Fig Difference between BHB1356 and control (Δ) for plant height, root lodging, stalk lodging, and stand count across multiple field trials, testers and locations. (DOCX)

S5 Fig Western-blots used in pull-down assays confirming 1:1 interaction of *ictB* with a subset of proteins. (DOCX)

S1 Table Details on field plot locations. (XLSX)

S2 Table Field results by trial. (XLSX)

S3 Table Protein-protein interaction details. (XLSX)

S4 Table Data repository. (XLSX)

S1 Material Data repository for protein-protein interaction. BHB-1 refers to the *ictB* fragment used in testing.
(7Z) Click here for additional data file.
