# Supplementary material for: Transgenic insertion of the cyanobacterial membrane protein ictB increases grain yield in Zea mays through increased photosynthesis and carbohydrate production
Source: PLoS One. 2021 Feb 4;16(2):e0246359. doi: 10.1371/journal.pone.0246359 (PMC7861388; doi:10.1371/journal.pone.0246359)
Supplement: S1 Fig — (DOCX) [file pone.0246359.s001.docx]

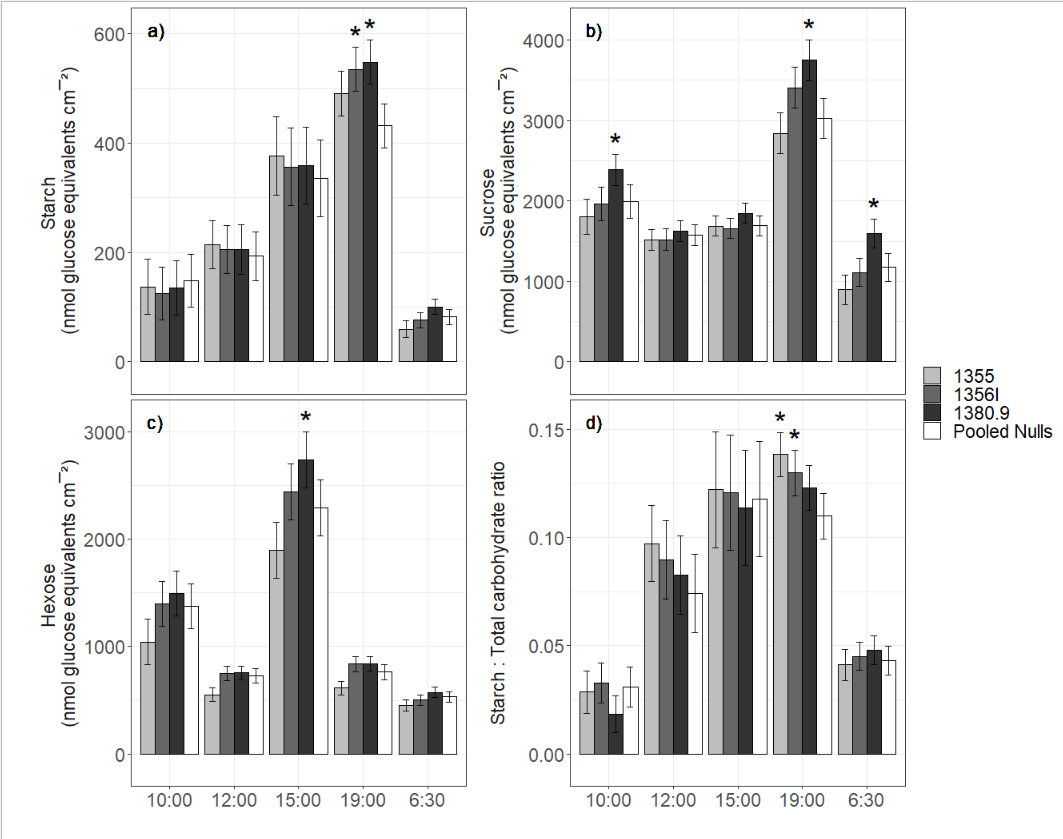


Supplementary Figure S1. Leaf carbohydrate content measured at different timepoints in a controlled-environment experiment including 3 transgenic ictB insertion events (1355, 1356 and 1380.9) and with pooled null transgenic plants used as control. a) starch, b) sucrose, c) hexose, d) starch:total carbohydrate ratio. Bars show the LSmean, error bars give the LSD05/2. * indicates significant difference (P<0.05) from a one-tailed two-sample t-test evaluating whether each event surpassed the control.
