# Supplementary material for: Transgenic insertion of the cyanobacterial membrane protein ictB increases grain yield in Zea mays through increased photosynthesis and carbohydrate production
Source: PLoS One. 2021 Feb 4;16(2):e0246359. doi: 10.1371/journal.pone.0246359 (PMC7861388; doi:10.1371/journal.pone.0246359)
Supplement: S2 Fig — (DOCX) [file pone.0246359.s002.docx]

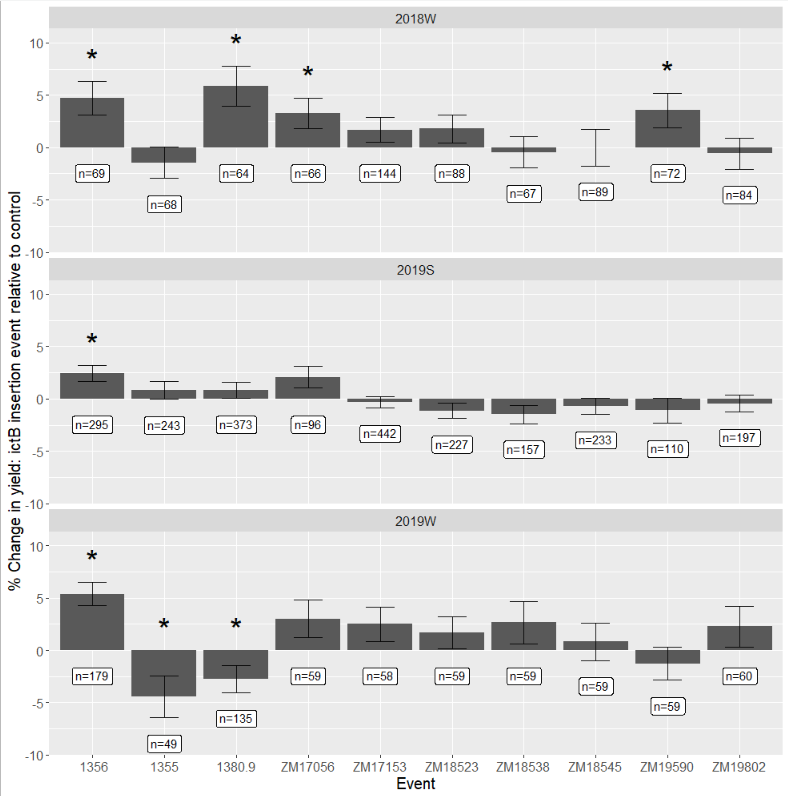


Supplementary Figure S2. % difference between ictB insertion event and control for grain yield, across multiple field trials, testers and locations, in 10 events including the lead event 1356. Bars are mean ± standard error. Numbers below bars give the number of plots used per event in each growing season (2018W, 2019S, 2019W). P-values are from a two-tailed t-test evaluating whether values are different from 0. * indicates significant difference (P<0.05) of the Δ (i.e. ictB event minus control) from 0 based on a two-tailed t-test.
