# Supplementary material for: Transgenic insertion of the cyanobacterial membrane protein ictB increases grain yield in Zea mays through increased photosynthesis and carbohydrate production
Source: PLoS One. 2021 Feb 4;16(2):e0246359. doi: 10.1371/journal.pone.0246359 (PMC7861388; doi:10.1371/journal.pone.0246359)
Supplement: S3 Fig — (DOCX) [file pone.0246359.s003.docx]

Supplementary Figure S3. *ictB* structure. The protein domains used in protein-protein interactions are outlined in red. The membrane is shown in orange.


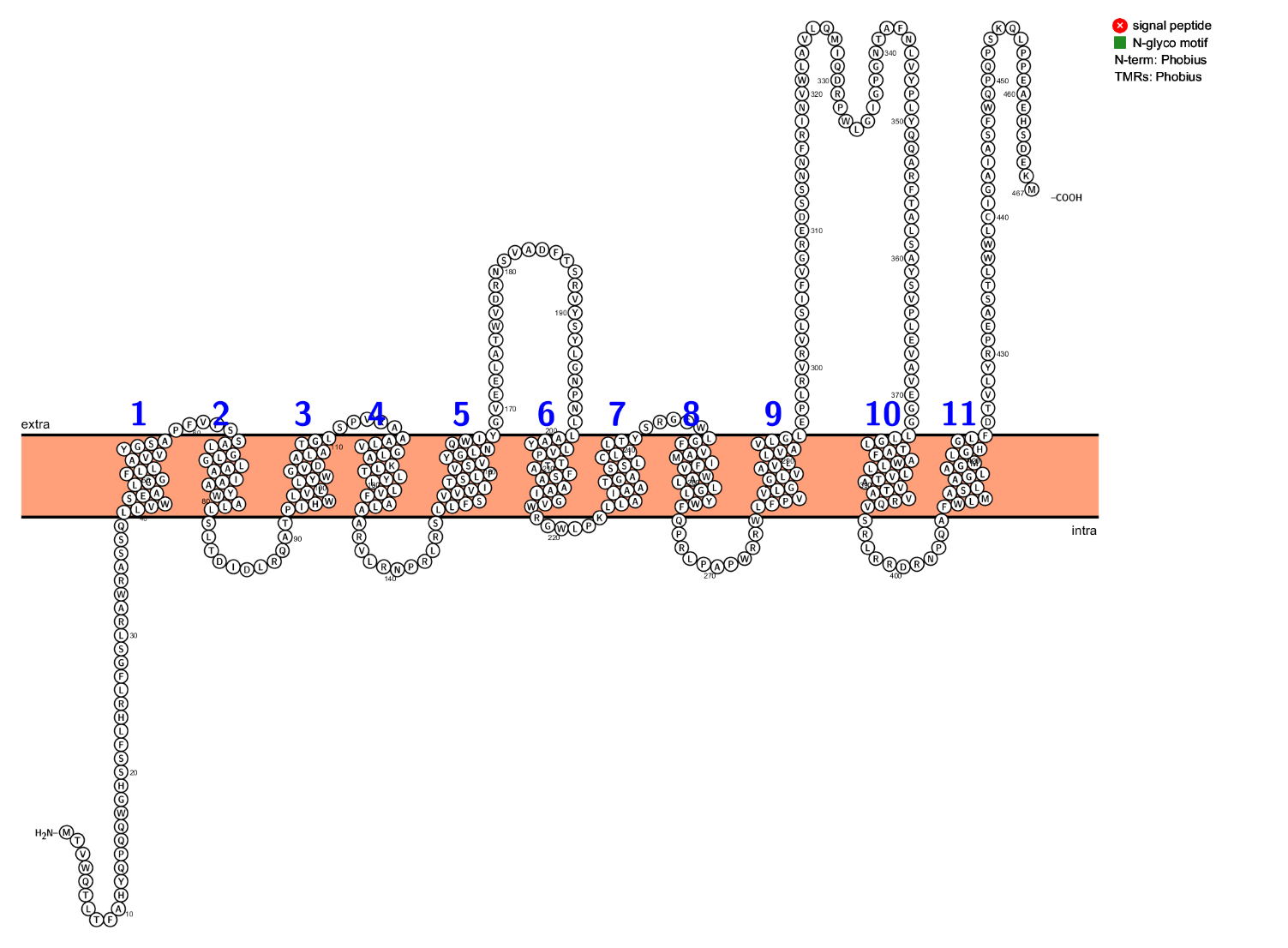


N-terminus

Big loop region

C-terminus
