# Supplementary material for: Transgenic insertion of the cyanobacterial membrane protein ictB increases grain yield in Zea mays through increased photosynthesis and carbohydrate production
Source: PLoS One. 2021 Feb 4;16(2):e0246359. doi: 10.1371/journal.pone.0246359 (PMC7861388; doi:10.1371/journal.pone.0246359)
Supplement: S4 Fig — (DOCX) [file pone.0246359.s004.docx]

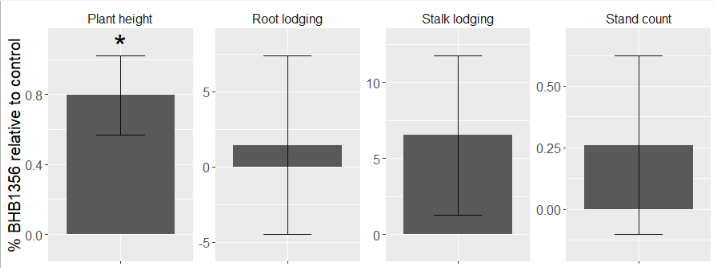


Supplementary Figure S4. % difference between BHB1356 and control for plant height, root lodging, stalk lodging, and stand count across multiple field trials, testers and locations. Bars are the mean ± standard error. * indicates significant difference (P<0.05) of the Δ (i.e. BHB1356 minus control) from 0 based on a two-tailed t-test.
