# Supplementary material for: Transgenic insertion of the cyanobacterial membrane protein ictB increases grain yield in Zea mays through increased photosynthesis and carbohydrate production
Source: PLoS One. 2021 Feb 4;16(2):e0246359. doi: 10.1371/journal.pone.0246359 (PMC7861388; doi:10.1371/journal.pone.0246359)
Supplement: S5 Fig — (DOCX) [file pone.0246359.s005.docx]

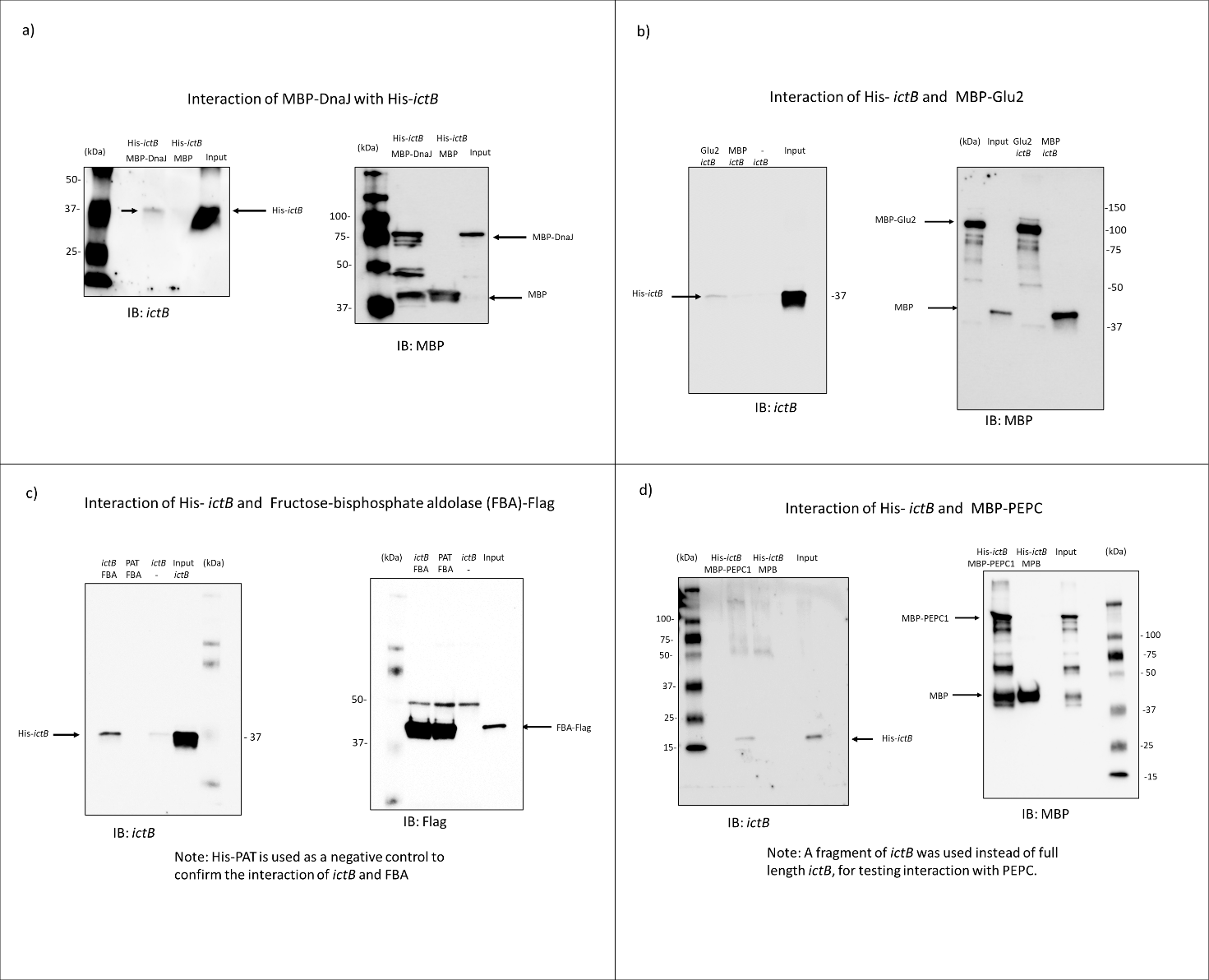


Supplementary Figure S5: Western-blots used in pull-down assays confirming 1:1 interaction of *ictB* with a) Molecular chaperone Hsp40/DnaJ family protein, b) beta-D-glucosidase precursor (glu2), c) Fructose-bisphosphate aldolase, d) phosphoenolpyruvate carboxylase
